# Impact of dose calculation accuracy on inverse linear energy transfer optimization for intensity‐modulated proton therapy

**DOI:** 10.1002/pro6.1179

**Published:** 2022-12-08

**Authors:** Mei Chen, Wenhua Cao, Pablo Yepes, Fada Guan, Falk Poenisch, Cheng Xu, Jiayi Chen, Yupeng Li, Ivan Vazquez, Ming Yang, X. Ronald Zhu, Xiaodong Zhang

**Affiliations:** ^1^ Department of Radiation Oncology Ruijin Hospital Shanghai Jiao Tong University School of Medicine Shanghai China; ^2^ Department of Radiation Physics The University of Texas MD Anderson Cancer Center Houston Texas USA; ^3^ Physics and Astronomy Department Rice University Houston Texas USA

**Keywords:** dose calculation accuracy, intensity‐modulated proton therapy, inverse optimization, linear energy transfer

## Abstract

**Objective:**

To determine the effect of dose calculation accuracy on inverse linear energy transfer (LET) optimization for intensity‐modulated proton therapy, and to determine whether adding more beams would improve the plan robustness to different dose calculation engines.

**Methods:**

Two sets of intensity‐modulated proton therapy plans using two, four, six, and nine beams were created for 10 prostate cancer patients: one set was optimized with dose constraints (DoseOpt) using the pencil beam (PB) algorithm, and the other set was optimized with additional LET constraints (LETOpt) using the Monte Carlo (MC) algorithm. Dose distributions of DoseOpt plans were then recalculated using the MC algorithm, and the LETOpt plans were recalculated using the PB algorithm. Dosimetric indices of targets and critical organs were compared between the PB and MC algorithms for both sets of plans.

**Results:**

For DoseOpt plans, dose differences between the PB and MC algorithms were minimal. However, the maximum dose differences in LETOpt plans were 11.11% and 15.85% in the dose covering 98% and 2% (D2) of the clinical target volume, respectively. Furthermore, the dose to 1 cc of the bladder differed by 11.42 Gy (relative biological effectiveness). Adding more beams reduced the discrepancy in target coverage, but the errors in D2 of the structure were increased with the number of beams.

**Conclusion:**

High modulation of LET requires high dose calculation accuracy during the optimization and final dose calculation in the inverse treatment planning for intensity‐modulated proton therapy, and adding more beams did not improve the plan robustness to different dose calculation algorithms.

## Graphical abstract

In this study, we investigate the dose calculation accuracy of intensity‐modulated proton therapy plans using novel linear energy transfer optimization. Our results showed linear energy transfer‐optimized intensity‐modulated proton therapy plans demand high calculation accuracy in inverse optimization, and adding more beams did not improve the plan robustness to dose calculation error. We recommend to use the same dose calculation algorithm in linear energy transfer optimization and final dose calculation.

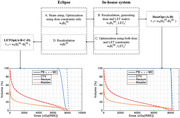

## 1 INTRODUCTION

As recommended by the International Commission on Radiation Units and Measurements, in clinical practice, proton therapy used a constant relative biological effectiveness (RBE) of 1.1, which assumes proton therapy is 10% more biologically effective than photon therapy.^1^ However, experimental studies have shown that proton RBE value varies with physical and radiobiological parameters, such as linear energy transfer (LET), dose per fraction, the α/β ratio of the tissues, and the biological endpoint.^2^, ^3^, ^4^ The use of biological optimization accounting for the variable RBE might have the potential to fully exploit the biological advantages of protons and improve the therapeutic index of proton therapy.

Owing to uncertainties in the radiobiological parameters and the discrepancy among different RBE calculation models, researchers proposed to optimize LET instead of the variable RBE‐weighted dose based on the fact that RBE increases with LET.^5^, ^6^, ^7^, ^8^, ^9^, ^10^, ^11^ The physical quantity of the averaged LET from the primary protons can be accurately predicted by Monte Carlo (MC) simulation or analytical modeling,^12^, ^13^, ^14^, ^15^, ^16^, ^17^ and modulated through planning in multi‐field optimization intensity‐modulated proton therapy (IMPT). Studies have shown that LET painting is an effective approach to boost the biological effects within the target and to overcome hypoxia, leading to increased tumor control probability.^18^, ^19^, ^20^, ^21^, ^22^ The rule of thumb in LET painting is to bring the elevated LET around the Bragg peak region into the target. This planning technique is transitioning from forward optimization^21^ to inverse optimization^10^ based on the dose and LET distributions generated from MC simulations.

The validated MC algorithm is generally considered as the most accurate method in dose calculation, and has been introduced to commercial treatment planning systems (TPSs).^23^, ^24^ However, in clinical practice, the analytical pencil beam (PB) algorithm is commonly used in IMPT dose calculation because of its comparable accuracy and fast calculation speed. Recent studies have used MC calculations to examine the accuracy of PB algorithms for IMPT.^24^, ^25^, ^26^ In the studies of clinical patient cases, dosimetric indices of the target agreed within 4% between PB and MC algorithms,^25^, ^26^ and a high discrepancy in dose was rarely reported.

Adding LET modulation to the conventional dose modulation could make the multi‐field optimization IMPT plan optimization more complex than before, posing a great challenge to the dose calculation engine. Two clinical trials of LET‐optimized IMPT have been conducted recently.^27^, ^28^ Before this advanced treatment planning technique is introduced into clinical practice, the dose calculation accuracy must be verified to ensure plan quality and patient safety. Currently, there is no LET optimizer with Food and Drug Administration approval available in the commercial TPSs. Therefore, plans using an MC‐based LET optimizer developed in‐house should be recalculated by the clinical TPS based on the PB dose calculation engine. However, the dose uncertainties of LET‐manipulated plans have been seldom investigated. It is of note that inaccurate dose calculation may result in the under‐dosage of the target and over‐dosage of the organs at risk (OARs), diminishing the benefits of advanced treatment planning. Therefore, it is imperative to investigate the discrepancy between dose distributions calculated using the PB and MC algorithms for LET‐optimized IMPT plans.

In the current study, we evaluated the dose differences between the two algorithms in calculating conventional dose‐optimized (DoseOpt) and LET‐optimized (LETOpt) IMPT plans for selected prostate cancer patients. Given the greater degree of freedom of the multi‐field approach in redistributing LET, the impact of the number of beams on the dose differences was also investigated. To the best of our knowledge, this is the first investigation of the impact of dose calculation accuracy on the inverse LET optimization for IMPT under the clinical scenarios with two different dose calculation engines involved in the optimization and final dose calculation.

## 2 METHODS

### 2.1 Patient selection and target definition

A total of 10 patients with localized prostate cancer receiving IMPT at The University of Texas MD Anderson Cancer Center were selected for the present retrospective study. Delineation of regions of interest and treatment planning was performed in Eclipse V13.7 (Varian Medical Systems) TPS. For IMPT planning, a scanning target volume (STV) was created by anisotropically expanding the clinical target volume (CTV). The STV‐CTV margin was 1.2 cm laterally, 0.5 cm in the superior and inferior directions, 0.6 cm anteriorly, and 0.4 cm posteriorly. The prescription was 78 Gy (RBE) in 39 fractions, assuming a constant RBE of 1.1.

### 2.2 Dose calculation engine

The Eclipse TPS has been commissioned for clinical use at our institution, and the proton PB convolution superposition algorithm is used to calculate the dose distribution.^29^ For dose‐averaged LET (LET_d_) calculation, we used a fast dose calculator (FDC) developed in‐house. FDC adopted a track‐repeating algorithm that uses the dataset generated using the general‐purpose Monte Carlo toolkit Geant4.^30^, ^31^, ^32^, ^33^, ^34^ FDC calculates the dose deposited in various materials by re‐tracking the particles in the dataset of the proton trajectories in water, after correcting for the stopping power and scattering angles. The RBE‐weighted dose reported by FDC is dose‐to‐water calculated with constant RBE. The accuracy of FDC has been validated against in‐water measurements of depth, and lateral profiles and full MC simulations (based on Geant4) of patient cases.^35^, ^36^

### 2.3 Treatment planning and optimization

**FIGURE 1:**
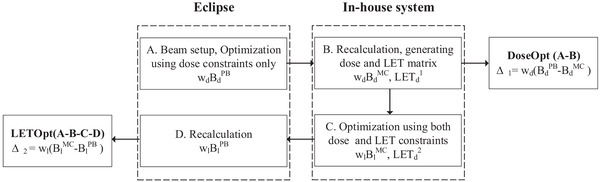
Schematic diagram of the workflow of treatment planning and dose comparison for the dose‐optimized (DoseOpt) and linear energy transfer‐optimized (LETOpt) plans. The dose of *x* plan is represented by w*_x_* B*_x_* ^A^, in which B*_x_* ^A^ is the beamlet given by A dose calculation engine and w*_x_* corresponds to the weight of the beamlet. LET_d_^N^ is the dose‐averaged LET in plan N

Figure 1 shows the workflow of creating and re‐calculating the DoseOpt and LETOpt plans. In the first step, we created four plans with two, four, six, and nine coplanar beam angles in Eclipse, setting the STV as the beam‐specific target for each beam (Table 1). An arrangement of two contralateral beams is the class solution for prostate cases in clinical practice (plan 1). Posterior oblique beams were added to avoid a high LET component in the rectum (plan 2), and anterior oblique beams were added to avoid a high LET component in the bladder (plan 3). To explore the potential benefit of proton arc therapy for the robustness of the dose calculation engine, nine evenly spaced beams were also created (plan 4). Plans were optimized using selective robust optimization based on the dose constraints only,^37^ in which eight scenarios, including setup and range uncertainties, were taken into account. The output dose distribution was denoted as $\omega_{d}B_{d}^{PB}$.

**TABLE 1:** Beam arrangement for each plan

		Beam angles (°)								
Plan #	No. beams	A	B	C	D	E	F	G	H	I
1	2	270	90							
2	4	270	90	140	220					
3	6	270	90	140	220	35	325			
4	9	20	60	100	140	180	220	260	300	340

In the second step, the DoseOpt plans were recalculated by FDC to generate the binary files containing the beamlet information for the subsequent LET_d_ optimization. At the same time, we obtained the MC‐calculated dose and LET_d_ distribution ($\omega_{d}B_{d}^{MC}$,$\;LET_{d}^{1}$) for the DoseOpt plans. In the third step, a LET‐incorporated optimization engine developed in‐house was used. The optimization applied a composite cost function including the dose deviation and the LET_d_ (maximization) in target and OARs (minimization).^10^ The optimal solution was represented by $\omega_{l}B_{l}^{MC}$ and$\;LET_{d}^{2}$. In the last step, the LETOpt plan was then imported into Eclipse and recalculated using the PB algorithm ($\omega_{l}B_{l}^{PB})$ for the clinical evaluation.

Optimization objectives were kept the same for each plan in either DoseOpt or LETOpt optimization. For the dosimetric criteria of MC‐calculated LETOpt plans, we allowed 2% variation in the equivalent uniform dose of CTV (*a* = −10), 2% or 2 Gy (RBE) increase in the equivalent uniform dose of rectum (*a* = 11.11) and bladder (*a* = 2), compared with PB‐calculated DoseOpt plans. The values of *a* were taken from previous studies. ^38^, ^39^ Nevertheless, both MC‐calculated LETOpt plans and the PB‐calculated DoseOpt plans are required to fulfill the planning directive (Supplementary Table S1).

### 2.4 analysis

LET_d_ distribution of the CTV was quantified using the mean LET_d_ and LET_d_ covering 98% of the CTV. Dose distributions calculated by the PB and MC algorithms were compared for the DoseOpt plans and the LETOpt plans. Dose difference (Δ_1_,$\;\Delta_{2}$) was quantified using the dosimetric indices of targets and OARs based on the dose–volume histogram. Target coverage was assessed in terms of the dose covering 98% (D98) and 2% (D2) of the CTV. The mean dose to and maximum dose to 1cc (D1cc) of the bladder and rectum were evaluated. The Wilcoxon signed‐rank test^40^ was performed with SPSS version 24.0 (IBM) to determine the differences in the dosimetric indices of plans calculated by the PB and MC algorithms, and the significance level was set at 0.05.

## 3 RESULTS

With regard to the dose distribution, the variation of MC‐calculated LETOpt plans from PB‐calculated DoseOpt plans was within the tolerance level (Supplementary Table S2). The enhancement of dose‐averaged LET (LET_d_) in the target by the LETOpt is presented in Table 2. The mean LET_d_ and the LET_d_ for 98% of the clinical target volume (CTV) were significantly increased (*p* < 0.001) from the DoseOpt plans to the LETOpt plans by a mean of 72.1% (range 59.8–85.6%) and 48.7% (range 42.4–57.2%), respectively. These results suggested that more high LET components were introduced in the targets of the LETOpt plans than in those of the DoseOpt plans.

**TABLE 2:** Indices of dose‐averaged linear energy transfer distribution in clinical target volume for the dose‐optimized and linear energy transfer‐optimized plans

	Mean LET_d_ of CTV (keV/μm)				LET_d_ to 98% of CTV (keV/μm)			
Plan	DoseOpt	LETOpt	Δ^*^	*p*‐value	DoseOpt	LETOpt	Δ^*^	*p*‐value
2‐field	2.6 ± 0.1	4.4 ± 0.4	69.9 ± 15.5%	<0.05	2.4 ± 0.1	3.4 ± 0.3	43.5 ± 11.5%	<0.05
4‐field	2.9 ± 0.1	4.6 ± 0.3	59.8 ± 7.0%	<0.05	2.5 ± 0.1	3.6 ± 0.3	42.4 ± 9.2%	<0.05
6‐field	2.8 ± 0.2	4.9 ± 0.4	73.1 ± 10.7%	<0.05	2.6 ± 0.2	3.9 ± 0.3	51.8 ± 11.4%	<0.05
9‐field	2.9 ± 0.2	5.4 ± 0.3	85.6 ± 5.1%	<0.05	2.6 ± 0.2	4.1 ± 0.3	57.2 ± 11.4%	<0.05

Abbreviations: CTV, clinical target volume; DoseOpt, dose‐averaged linear energy transfer; LET_d_, dose‐averaged linear energy transfer; LETOpt, linear energy transfer‐optimized.

*Δ is the percentage difference in LET_d_ between the LETOpt and DoseOpt plans.

**FIGURE 2:**
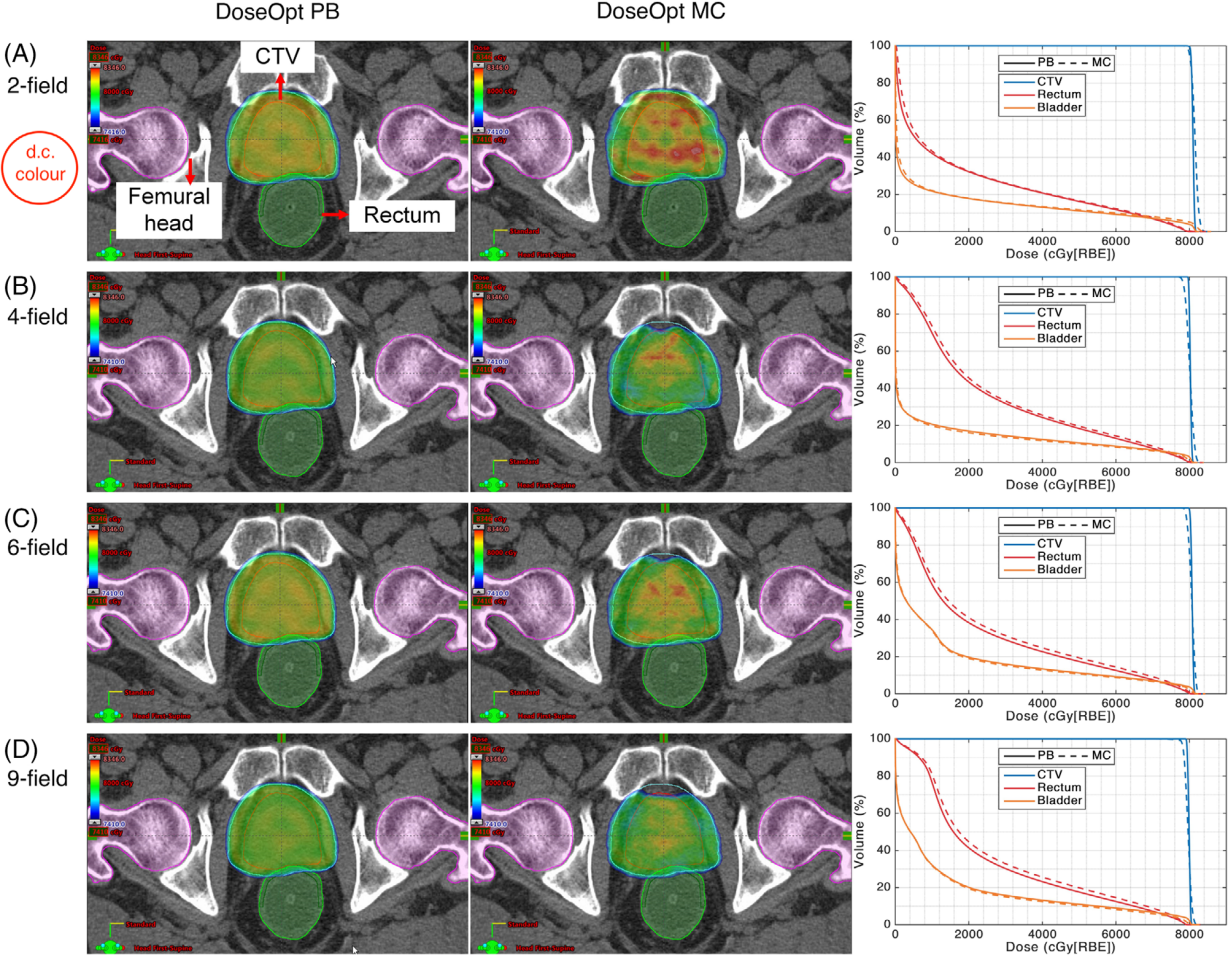
(Left to right columns) Pencil beam (PB)‐calculated dose distribution in the transverse plane, Monte Carlo (MC)‐calculated dose distribution in the transverse plane, and comparison of dose‐volume histograms for dose‐optimized (DoseOpt) plans using (a) two‐field, (b) four‐field, (c) six‐field, and (d) nine‐field arrangements. CTV, clinical target volume

**FIGURE 3:**
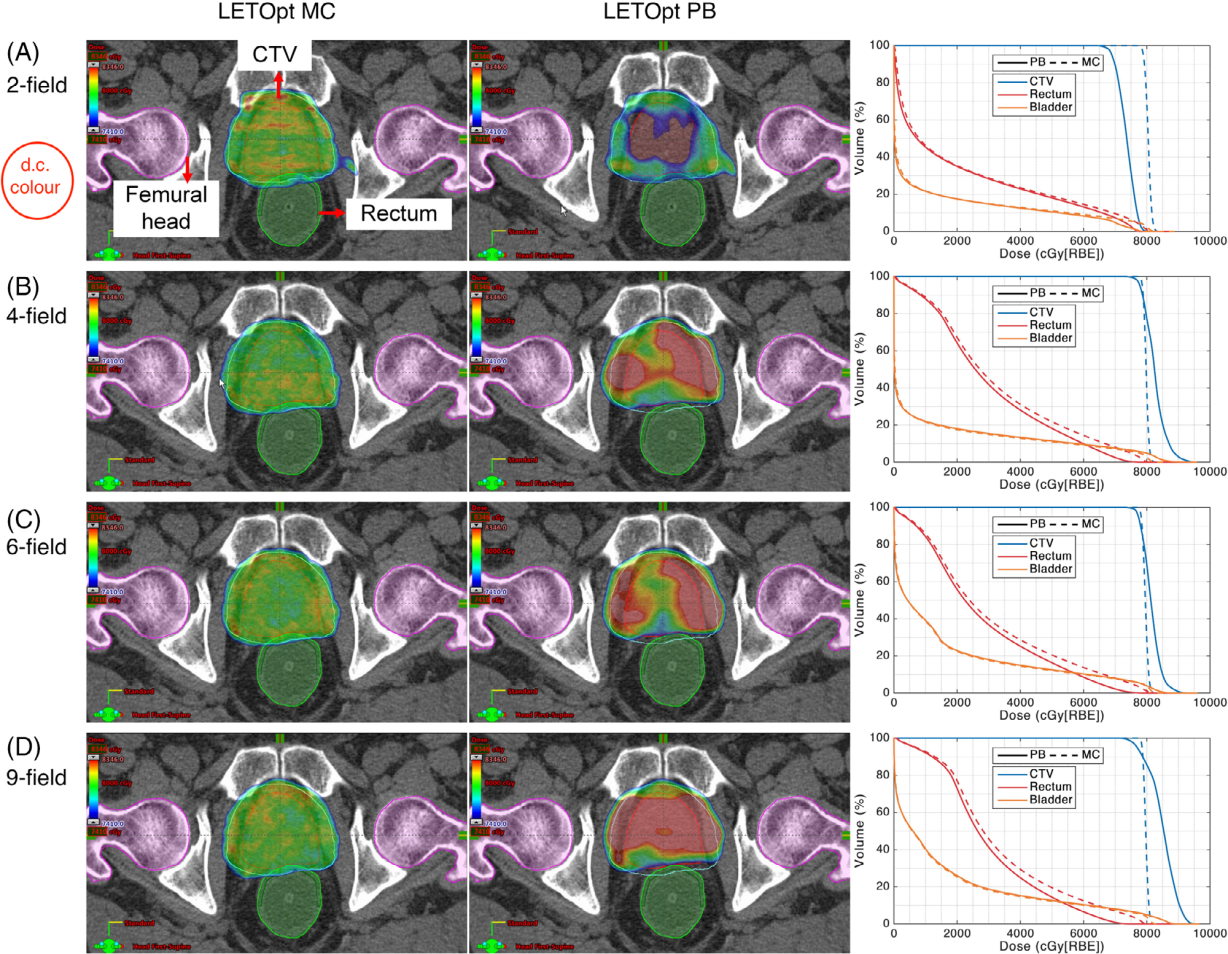
(Left to right columns) Pencil beam (PB)‐calculated dose distribution in the transverse plane, Monte Carlo (MC)‐calculated dose distribution in the transverse plane, and comparison of dose–volume histograms for linear energy transfer‐optimized (LETOpt) plans using (a) two‐field, (b) four‐field, (c) six‐field, and (d) nine‐field arrangements. CTV, clinical target volume

**FIGURE 4:**
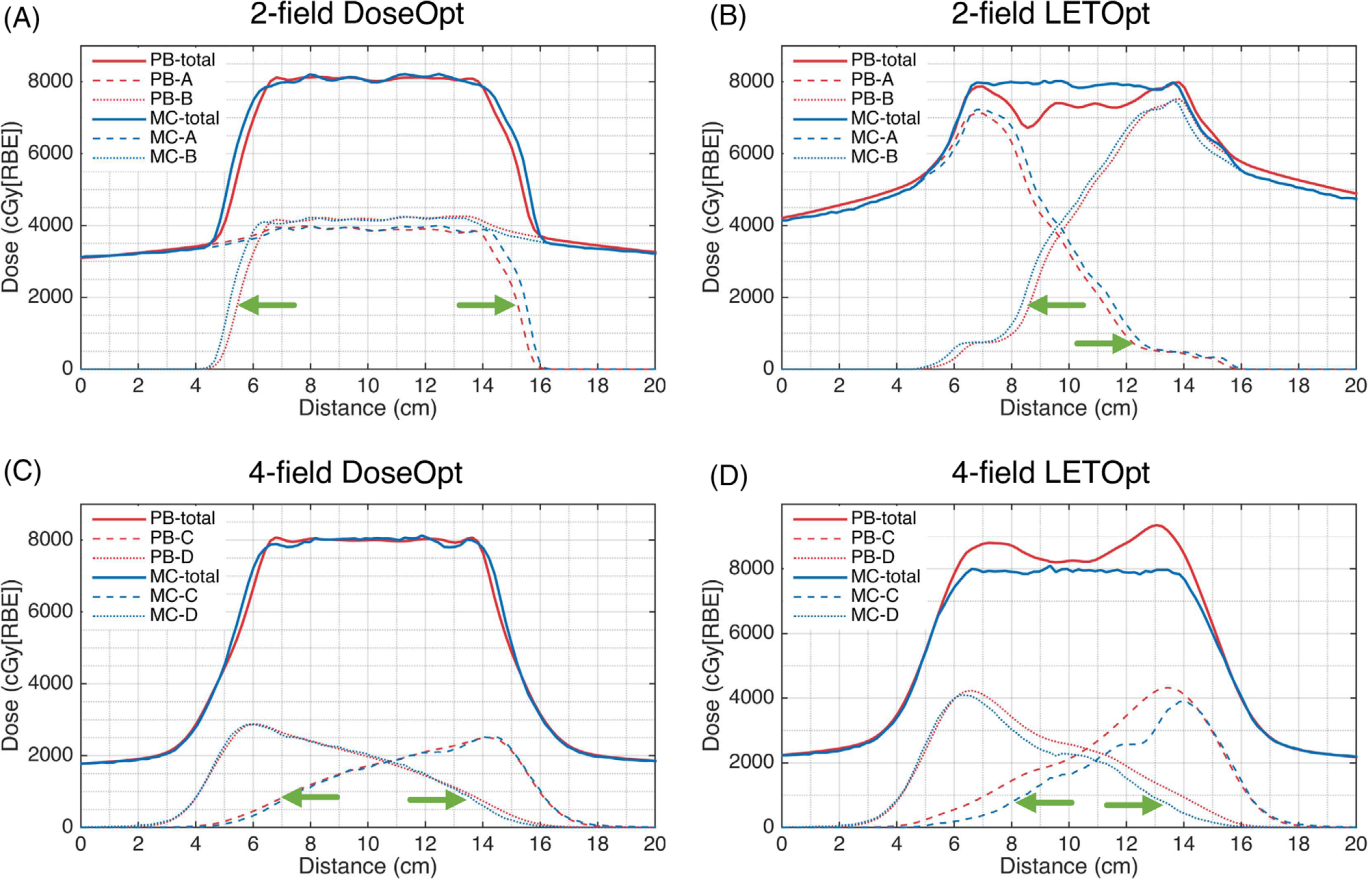
Comparison of dose profiles along the mid–scanning target volume line between dose‐optimized (DoseOpt) and linear energy transfer‐optimized (LETOpt) optimization techniques for two‐field and four‐field plans. The angles of the fields are (a) 270°, (b) 90°, (c) 140°, and (d) 220°, respectively. MC, Monte Carlo; PB, pencil beam

Figures 2 and 3 show the dose distributions and dose–volume histograms of the DoseOpt and LETOpt plans calculated by the PB and MC algorithms for a representative case. Overall, the dose distributions became less homogenous when the PB‐computed plans were recalculated by the MC algorithm and vice versa. As shown in the dose profiles of the two‐field and four‐field plans along the mid‐STV line (Figure 4), each beam stops at the distal edge of the target in the DoseOpt plan, whereas the distal end of each beam stops within the CTV in the LETOpt plan. Notably, the DoseOpt optimization generated flat fields across the target in the beam direction, whereas the LETOpt optimization created ramp‐like fields patching with each other at the center of the target. For the DoseOpt plans, the PB algorithm and MC algorithm yielded similar dose distributions in terms of the target coverage. Of note, for the two‐field LETOpt plan, the target was significantly under‐dosed in the PB calculation compared with the MC calculation. For the DoseOpt plans, a slight dose difference between algorithms was observed at the periphery of the target, whereas for the LETOpt plans, a substantial dose difference between algorithms was observed near the field patching area within the target.

Tables 3 and 4 summarize the key indices of the doses calculated by PB and MC algorithms for the DoseOpt and LETOpt plans. For the DoseOpt plans, the maximum deviation in the dosimetric indices of the CTV between the PB and MC calculations was limited to 2.6%. The dose to 1 cc (D1cc) of the rectum differed between the two algorithms by a mean of 1.3% (1 Gy [RBE]) (range 0.8–1.9%). The difference in the mean dose of the bladder was less than 0.5 Gy (RBE).

**TABLE 3:** Dosimetric indices of the dose‐averaged linear energy transfer plans calculated by pencil beam and Monte Carlo algorithms

		CTV		Rectum		Bladder	
Plan		D98 (Gy(RBE))	D2 (Gy[RBE])	D1cc (Gy[RBE])	Dmean (Gy[RBE])	D1cc (Gy[RBE])	Dmean (Gy[RBE])
2‐field	PB	79.1 ± 0.5	80.5 ± 0.5	79.3 ± 0.5	25.3 ± 3.4	80.8 ± 0.6	12.1 ± 4.0
	MC	77.5 ± 1.0	81.6 ± 0.8	80.4 ± 1.0	25.7 ± 3.3	82.9 ± 1.0	12.5 ± 4.1
	Δ* (%)	−2.1 ± 0.7	1.3 ± 0.6	1.4 ± 0.6	1.8 ± 1.0	2.6 ± 0.6	3.4 ± 1.2
	*p*‐value	<0.05	<0.05	<0.05	<0.05	<0.05	<0.05
4‐field	PB	79.2 ± 0.3	80.5 ± 0.3	79.3 ± 0.6	31.1 ± 3.5	80.8 ± 0.3	11.7 ± 3.7
	MC	77.1 ± 0.7	81.6 ± 0.4	80.8 ± 0.9	32.5 ± 3.9	82.5 ± 0.6	11.3 ± 4.1
	Δ* (%)	−2.6 ± 0.7	1.3 ± 0.4	1.9 ± 0.9	4.6 ± 1.7	2.0 ± 0.8	−4.4 ± 3.9
	*p*‐value	<0.05	<0.05	<0.05	<0.05	<0.05	0.06
6‐field	PB	79.3 ± 0.4	80.6 ± 0.4	79.2 ± 0.6	27.7 ± 3.1	80.9 ± 0.5	14.4 ± 3.3
	MC	77.3 ± 0.8	81.2 ± 0.4	79.9 ± 1.0	29.1 ± 3.2	81.9 ± 0.4	14.0 ± 3.6
	Δ* (%)	−2.5 ± 0.7	0.7 ± 0.4	0.8 ± 1.1	5.1 ± 1.6	1.2 ± 0.2	−2.9 ± 2.2
	*p*‐value	<0.05	<0.05	<0.05	<0.05	<0.05	<0.05
9‐field	PB	79.2 ± 0.4	80.5 ± 0.3	79.1 ± 0.5	29.9 ± 3.3	80.8 ± 0.4	14.9 ± 3.4
	MC	77.1 ± 0.6	81.4 ± 0.5	79.8 ± 0.8	31.4 ± 3.4	82.1 ± 1.0	14.3 ± 3.5
	Δ* (%)	−2.6 ± 0.6	1.1 ± 0.2	0.9 ± 1.1	5.0 ± 1.5	1.5 ± 1.2	−3.6 ± 2.0
	*p*‐value	<0.05	<0.05	<0.05	<0.05	<0.05	<0.05

Abbreviations: CTV, clinical target volume; D1cc, maximum dose to 1 cc of the volume; D2, dose covering 2% of the volume; D98, dose covering 98% of the volume; Dmean, mean dose; MC, Monte Carlo; RBE, relative biological effectiveness; PB, pencil beam; Vp, coverage of the volume by the prescribed dose.

* Δ is the percentage difference in the dose indices between the MC‐computed and PB‐computed dose‐averaged linear energy transfer plans.

**TABLE 4:** Dosimetric indices of the linear energy transfer‐optimized plans calculated by pencil beam and Monte Carlo algorithms

		CTV		Rectum		Bladder	
Plan		D98 (Gy[RBE])	D2 (Gy[RBE])	D1cc (Gy[RBE])	Dmean (Gy[RBE])	D1cc (Gy[RBE])	Dmean (Gy[RBE])
2‐field	MC	78.3 ± 0.2	82.1 ± 0.3	81.8 ± 0.5	26.3 ± 3.1	83.5 ± 0.4	11.9 ± 4.1
	PB	69.6 ± 2.7	79.0 ± 1.3	78.5 ± 0.5	25.0 ± 3.0	79.2 ± 1.5	11.2 ± 4.1
	Δ* (%)	−11.1 ± 3.4	−3.7 ± 1.7	−4.1 ± 0.9	−5.0 ± 1.4	−5.2 ± 1.7	−6.2 ± 2.2
	*p*‐value	<0.05	<0.05	<0.05	<0.05	<0.05	<0.05
4‐field	MC	78.2 ± 0.2	81.5 ± 0.2	81.2 ± 0.6	35.7 ± 2.7	82.8 ± 0.6	11.6 ± 4.0
	PB	74.2 ± 2.0	90.1 ± 4.8	77.0 ± 1.8	32.7 ± 2.4	91.8 ± 5.3	12.3 ± 3.6
	Δ* (%)	−5.1 ± 2.6	10.6 ± 6.0	−5.2 ± 2.3	−8.5 ± 3.0	10.8 ± 6.2	7.1 ± 5.5
	*p*‐value	<0.05	<0.05	<0.05	<0.05	<0.05	0.06
6‐field	MC	78.2 ± 0.2	81.2 ± 0.2	81.2 ± 0.7	32.9 ± 2.9	82.7 ± 0.6	14.7 ± 3.9
	PB	74.9 ± 1.1	91.6 ± 6.8	77.7 ± 1.8	30.0 ± 2.4	91.0 ± 5.8	15.3 ± 3.5
	Δ* (%)	−4.2 ± 1.5	12.7 ± 8.3	−4.3 ± 2.6	−9.0 ± 3.1	10.0 ± 7.1	4.4 ± 3.6
	*p*‐value	<0.05	<0.05	<0.05	<0.05	<0.05	<0.05
9‐field	MC	78.3 ± 0.2	81.2 ± 0.3	80.9 ± 0.7	35.3 ± 2.9	82.3 ± 0.9	15.0 ± 3.6
	PB	73.0 ± 1.5	94.0 ± 7.3	74.9 ± 2.9	32.1 ± 2.4	93.7 ± 6.9	15.8 ± 3.4
	Δ* (%)	−6.7 ± 1.8	15.9 ± 9.0	−7.5 ± 3.3	−9.0 ± 3.3	13.9 ± 8.2	5.6 ± 3.9
	*p*‐value	<0.05	<0.05	<0.05	<0.05	<0.05	<0.05

Abbreviations: CTV, clinical target volume; D1cc, maximum dose to 1 cc of the volume; D2, dose covering 2% of the volume; D98, dose covering 98% of the volume; Dmean, mean dose; PB, pencil beam; MC, Monte Carlo; RBE, relative biological effectiveness; Vp, coverage of the volume by the prescribed dose;.

* Δ is the percentage difference in the dose indices between the PB‐computed and MC‐computed linear energy transfer‐optimized plans.

However, pronounced dose differences between algorithms were observed in the LETOpt plans. For target coverage, PB calculations compared with MC calculations showed degradation in the D98 of CTV at 11.1 ± 3.4%, 5.1 ± 2.6%, 4.2 ± 1.5%, and 6.7 ± 1.8% for the two‐field, four‐field, six‐field, and nine‐field plans, respectively. The D2 of CTV calculated by the PB algorithm was underestimated, relative to that of the MC algorithm, by 3.7 ± 1.7% for the two‐field plan, but was severely overestimated by 10.6 ± 6.0%, 12.7 ± 8.3% and 15.9 ± 9.0% for the other three plans. The dose difference between the PB and MC calculations of D1cc of the rectum was as large as 7.5 ± 3.3% (6.0 ± 2.7 Gy [RBE]) in the nine‐field plan. The maximum deviation in the D1cc of the bladder 13.9 ± 8.2% (11.4 ± 6.7 Gy [RBE]) was also found in the nine‐field plan.

## 4 DISCUSSION

The current study sought to verify the dose calculation accuracy of LET‐optimized IMPT. We focused on a type of LET manipulation known as the LET painting technique, whose priority is to deliver high LET components within the target. Our results showed a substantially larger difference in dose between the PB and MC algorithms for the LETOpt plans than for the conventional DoseOpt plans. For the LETOpt plans, the maximum dose differences between algorithms were 11.1% in the D98 of CTV, 15.9% in the D2 of CTV, and 11.4 Gy (RBE) in the D1cc of the bladder, whereas the differences in the same indices for the DoseOpt plans were 2.6%, 1.3%, and 2.1 Gy (RBE), respectively. This significant dose discrepancy between two dose calculation engines for a relatively homogeneous treatment site warrants the careful use of LET‐optimized IMPT plans with a high degree of intensity modulation.

For the DoseOpt plans, PB calculations agreed well with MC calculations, which was consistent with previous studies.^25^, ^26^ By using CTV‐based robust optimization,^37^, ^41^ the optimizer tended to limit the dose coverage of the beam that was more sensitive to uncertainties than other beams were and to avoid a sharp dose gradient in the target region. Thus, the dose distribution in the target of each beam was globally uniform along the beam direction (Figure 4A) and regionally uniform in the transverse direction (Figure 3 in Reference^41^). The difference between the PB and MC algorithms in the flat dose region was minimal (Figure 4A), whereas a distinct difference has been observed in the distal fall‐off region.^42^ In our DoseOpt plans, the difference in the distal region between the two algorithms was spread out as the beams stop at the periphery of the target. As such, we did not observe a great discrepancy in the dosimetric indices of the target and OARs.

In contrast, the LETOpt plans manifested dose differences between the two algorithms due to the distal field patching and the less uniform dose distribution of the ramp‐like fields. Low‐energy protons have higher LET_d_ than do the high‐energy protons. Therefore, the contribution of the relatively high‐energy protons was penalized during the optimization to increase LET_d_ in the target (Figure 4), resulting in more small fields near the distal end of the beam than in the DoseOpt plans. PB dose calculation calibrated under uniform dose distribution with broad beams might be inaccurate for the narrow beams because of the imperfect low‐dose halo modeling. Furthermore, the energy loss dispersion of protons (range straggling) was not well taken into account in the PB algorithm.^42^, ^43^ However, the irregular spot arrangement and range straggling can be better handled by the MC dose calculation, mainly because it tracks the interaction of every particle with the medium. The plan with the conventional arrangement of two contralateral beams showed the largest difference between algorithms in target coverage, in which the distal fall‐off from both beams converged. In this case, the plan was extremely sensitive to the uncertainty in the distal‐end modeling of the algorithm. The dose difference between the two algorithms also depended on the heterogeneity along the beam path. The results for the four‐field LETOpt plan (Figures 2 and 3, and Table 4) indicated that PB underestimated the dose for the two contralateral beams, but overestimated the dose for the oblique beams, and the overestimation outperformed the underestimation. With regard to the OAR protection, the LETOpt treatment planning compared with the DoseOpt planning also increased the difference between the two algorithms in the dose to rectum and bladder.

In addition to the plan robustness against different dose calculation engines, the robustness of the LETOpt plans against the setup and range uncertainties was also a clinical concern. We evaluated the robustness of the conventional and LET‐optimized plans, in which eight perturbation scenarios were taken into account. As it is shown in Figure S1, CTV in the LET‐optimized two‐field plan was not robust to range uncertainty as that was in the conventional plan. Bai et al.^44^ found that LET optimization incorporated with robust optimization could reduce the biological hot spots to the normal tissues near the target, and in the present study, we found that robust optimization is contradicting to LET optimization for target. Robust optimization tends to create fields that are flat in the target region. The flat fields reduced the high LET components in the target. In contrast, LET optimization for LET enhancement in target tends to create ramp fields that are not robust to range uncertainties. Regarding large underdosage and overdosage in the target volume, the LET‐optimized plans should be used with caution.

In inverse treatment planning, inaccurate or imprecise dose calculation introduced systematic error and convergence error.^45^, ^46^ MC dose calculation, if validated experimentally, is generally considered more accurate than the analytic algorithms in the dose calculation. However, we should be aware that MC dose calculation was limited with its statistical uncertainty. The acceptable value of statistical uncertainty is less than 2% in inverse treatment planning for voxelized computed tomography geometry.^47^ Jeraj and Keall showed that in inverse treatment planning, dose calculation free of statistical uncertainty was equivalent to MC dose calculation with regard to the optimization solutions if the dose errors were lower than the MC statistical uncertainty.^46^ Therefore, the accuracy of PB dose calculation might be adequate in inverse treatment planning for IMPT under conventional clinical beam settings, but not for IMPT under distal field patching. The discrepancy shown in the LET‐optimized plans indicated the significant convergence errors of PB dose calculation if it was used in the inverse IMPT treatment planning to find the optimal solution, such as the ramp‐like fields. In other words, the optimized weights of the beamlets given by PB dose calculation significantly diverge from the optimal solutions.

Furthermore, in the presence of convergence errors, it is unfeasible to create a clinically acceptable LET‐favored plan with ramp‐like fields using the current PB dose calculation in both forward and inverse treatment planning. Ensuring the accuracy of dose calculation is an important part of the patient‐specific quality assurance program, and a different algorithm is required to perform a secondary check. In the clinics that adopt the PB algorithm in the treatment planning, the MC algorithm is commonly used as an independent check for dose calculation accuracy. Another clinical scenario is using the MC algorithm during the final dose calculation in the TPS that has both MC and PB algorithms. The LET‐optimized plans presented in the study, if the PB algorithm was used in dose calculation, could fail a plan check in either scenario.

Although the issues raised in the present study are specific to the current framework in the clinics, and although the dose uncertainties could be reduced with fast MC‐based inverse treatment planning and final dose calculation in the future, the sensitivity of LET modulation to dose calculation errors persists, and the quality assurance for LET‐optimized IMPT plans is warranted.

## 5 CONCLUSION

In conclusion, high modulation of LET requires high calculation accuracy of physical quantities in inverse treatment planning for IMPT. Slight differences in the distal fall‐off modeling could cause significant dose error under the circumstance of distal field patching, and the dose error could not be washed out by adding more beams. MC or improved PB algorithms are preferable, and the same dose calculation engine is recommended to be used in both the LET optimization and the final dose calculation.

## CONFLICT OF INTEREST

The authors declare that they have read the article and there are no competing interests.

## Supporting information

Supporting information
