# Supplementary material for: Impact of dose calculation accuracy on inverse linear energy transfer optimization for intensity‐modulated proton therapy
Source: Precis Radiat Oncol. 2022 Dec 8;7(1):36–44. doi: 10.1002/pro6.1179 (PMC11935249; doi:10.1002/pro6.1179)
Supplement: Supplementary file 1 — Supporting information [file PRO6-7-36-s001.docx]

### Supplementary materials

**Table S1.** Planning directive for prostate cancer

|  | Clinical goal | |
| --- | --- | --- |
| Region of interest | Per protocol | Acceptable |
| STV | V78 ≥ 98% |  |
| Rectum | V80 ≤ 3%  V70 ≤ 20%  V60 ≤ 40%  V40 ≤ 60% |  |
| Bladder | V70 ≤ 20% | V70 ≤ 30% |

STV, scanning target volume; V*x*, relative volume of region receiving a dose of ≥*x* Gy(RBE).

**Table S2.** EUD variations of MC-calculated LETOpt plans from the PB-calculated DoseOpt plans

| Region of interest |  | 2-field  Gy (RBE) | 4-field  Gy (RBE) | 6-field  Gy (RBE) | 9-field  Gy (RBE) |
| --- | --- | --- | --- | --- | --- |
| CTV | DoseOpt | 79.8 ± 0.5 | 79.8 ± 0.3 | 79.9 ± 0.4 | 79.8 ± 0.3 |
|  | LETOpt | 80.1 ± 0.2 | 79.8 ± 0.2 | 79.6 ± 0.1 | 79.7 ± 0.2 |
|  | Δ^*^ (%) | 0.4 ± 0.6 | -0.1 ± 0.4 | -0.3 ± 0.6 | -0.2 ± 0.4 |
|  | *p*-value | 0.05 | 0.45 | 0.07 | 0.07 |
| Rectum | DoseOpt | 62.1 ± 1.1 | 62.3 ± 1.1 | 61.8 ± 1.2 | 61.8 ± 1.4 |
|  | LETOpt | 63.1 ± 1.1 | 62.3 ± 1.2 | 61.9 ± 1.3 | 61.8 ± 1.2 |
|  | Δ^*^ (%) | 1.6 ± 1.0 | -0.1 ± 0.9 | 0.2 ± 0.7 | 0.0 ± 0.6 |
|  | *p*-value | <0.05 | 0.80 | 0.65 | 0.72 |
| Bladder | DoseOpt | 25.7 ± 4.3 | 25.3 ± 4.2 | 26.9 ± 3.7 | 27.1 ± 3.7 |
|  | LETOpt | 25.3 ± 4.6 | 25.0 ± 4.5 | 27.0 ± 4.3 | 27.1 ± 4.0 |
|  | Δ^*^ (%) | -2.0 ± 2.1 | -1.2 ± 1.8 | 0.0 ± 3.4 | -0.1 ± 3.2 |
|  | *p*-value | <0.05 | 0.24 | 0.96 | 0.39 |

* Δ is the percentage difference in the dose indices between the MC-computed LETOpt plans and the PB-computed DoseOpt plans.

EUD, equivalent uniform dose; MC, Monte Carlo; PB, pencil beam; RBE, relative biological effectiveness; CTV, clinical target volume.


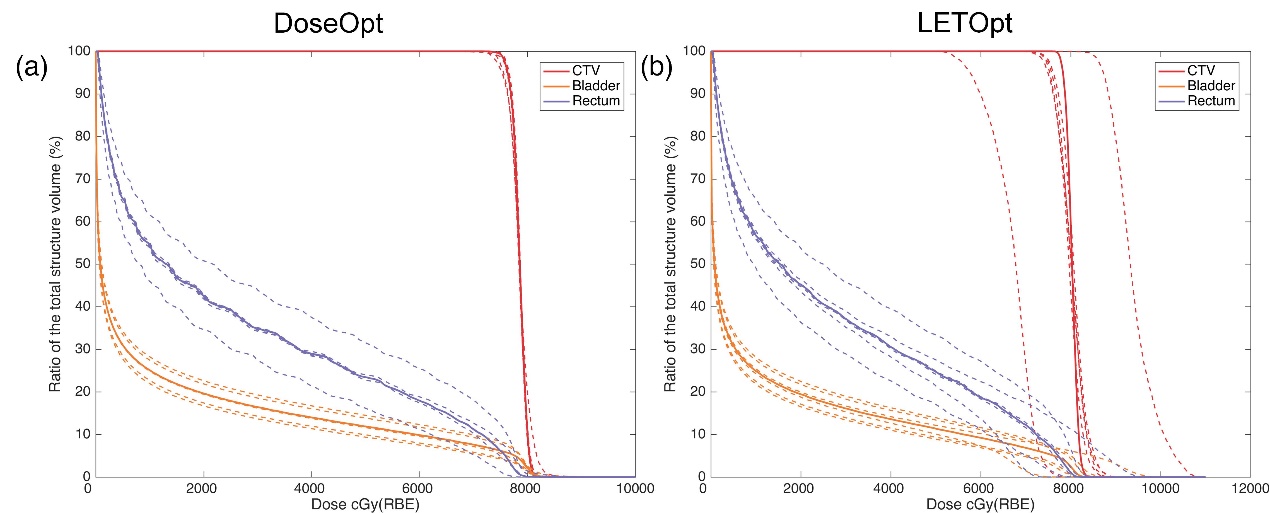


Figure S1 Dose volume histograms (DVHs) under eight uncertainty scenarios for the DoseOpt plan (a) and the LETOpt plan (b) with 2 fields. The solid lines represent the nominal plan and the dashed lines represent the perturbed plans.
